# Transcriptional Biomarkers of Steroidogenesis and Trophoblast Differentiation in the Placenta in Relation to Prenatal Phthalate Exposure

**DOI:** 10.1289/ehp.0900788

**Published:** 2009-09-15

**Authors:** Jennifer J. Adibi, Robin M. Whyatt, Russ Hauser, Hari K. Bhat, Barbara J. Davis, Antonia M. Calafat, Lori A. Hoepner, Frederica P. Perera, Deliang Tang, Paige L. Williams

**Affiliations:** 1 Department of Environmental Health, Harvard School of Public Health, Boston, Massachusetts, USA; 2 Columbia Center for Children’s Environmental Health, Mailman School of Public Health, New York, New York, USA; 3 Millennium Pharmaceuticals, Cambridge, Massachusetts, USA; 4 National Center for Environmental Health, Centers for Disease Control and Prevention, Atlanta, Georgia, USA; 5 Department of Biostatistics, Harvard School of Public Health, Boston, Massachusetts, USA

**Keywords:** epidemiology, gene expression, phthalates, placenta, pregnancy, prenatal, steroidogenesis, trophoblast differentiation

## Abstract

**Background:**

Phthalates can alter steroidogenesis and peroxisome proliferator–activated receptor gamma (PPARγ)–mediated transcription in rodent tissues. The placenta offers a rich source of biomarkers to study these relationships in humans.

**Objective:**

We evaluated whether gestational phthalate exposures in humans were associated with altered human placental steroidogenesis and trophoblast differentiation as measured by markers of mRNA transcription.

**Methods:**

We measured seven target genes in placentas collected from 54 Dominican and African-American women at delivery in New York City using quantitative real-time polymerase chain reaction (qPCR), normalized to 18S rRNA. qPCR results for the target genes were log-transformed, converted to *Z*-scores, and grouped into two functional pathways: steroidogenesis (aromatase, cholesterol side chain cleavage enzyme, 17β-hydroxysteroid dehydrogenase type 1, and cytochrome P450 1B1) and trophoblast differentiation (*PPAR*γ, aryl hydrocarbon receptor, and human chorionic gonadotropin). Repeated measures models were used to evaluate the association of phthalate metabolites measured in third-trimester urine samples with each group of target genes, accounting for correlation among the genes within a pathway.

**Results:**

Higher urinary concentrations of five phthalate metabolites were associated with lower expression of the target genes reflecting trophoblast differentiation. Results were less consistent for genes in the steroidogenesis pathway and suggested a nonlinear dose–response pattern for some phthalate metabolites.

**Conclusions:**

We observed a significant association between prenatal exposure to phthalates and placental gene expression within two pathways. Further studies are warranted to understand the significance of this association with respect to fetal development and placental function.

Phthalates are synthetic chemicals used in plastics, personal care products, and building materials. Phthalates are metabolized and excreted in urine 12–48 hr after exposure (Koch et al. 2005). Urinary phthalate metabolites measured have been used extensively as biomarkers of human exposure (Hauser and Calafat 2005).

The metabolites of at least four phthalates can induce a wide range of responses with potential relevance to human reproduction and development (Hauser and Calafat 2005; Swan 2008). When given to rats *in utero*, di(2-ethylhexyl) phthalate (DEHP), di-*n*-butyl phthalate (DnBP), diisobutyl phthalate (DiBP), and butylbenzyl phthalate (BBzP) altered steroidogenesis and produced a phenotype that involved reduced testosterone synthesis in fetal Leydig cells and concomitant effects on the male offspring resembling the testicular dysgenesis syndrome (Barlow et al. 2003; Ema et al. 2000; Mylchreest et al. 2000; Saillenfait et al. 2008; Sharpe 2001). Similarly, in female rats, DEHP, DnBP, and DiBP produced alterations in ovarian steroidogenesis, partly mediated by CYP19 (aromatase) inhibition and progesterone inhibition (Boberg et al. 2008; Davis et al. 1994a, 1994b; Lovekamp and Davis 2001).

The peroxisome proliferator–activated receptor protein gamma (PPARγ) is a master regulator of several reproductive and developmental pathways, including steroidogenesis, differentiation, fatty acid uptake and transport, and inflammation related to parturition (Froment et al. 2006; Schaiff et al. 2006). PPARγ is expressed in the human trophoblast and is essential to basic placental development and function (Fournier et al. 2007). Agonism of PPARγ by phthalate metabolites has been proposed as a mechanism of action (Lovekamp-Swan et al. 2003; Maloney and Waxman 1999). *In vivo* prenatal exposure to a metabolite of DEHP resulted in dose-dependent activation of PPARγ in rat placenta and changes in the expression of its downstream targets, including fatty acid transport protein and cytochrome oxidase-2 (Xu et al. 2008).

Prenatal exposure to DEHP has been associated with the timing of labor in four different cohorts, suggesting phthalate-related disruption in placental function (Adibi et al. 2009a; Latini et al. 2003; Whyatt et al. 2009; Wolff et al. 2008). We hypothesized that phthalate exposure could also be associated with alterations in human placental development and function, through PPARγ-mediated pathways (e.g., trophoblast differentiation).

We selected specific gene targets unified in two pathways based on their strong association with placental health and function and direct experimental evidence of their disruption by DEHP, DnBP, DiBP, or BBzP. Our criteria included targets downstream of those disrupted by phthalates and/or hypothesized to be disrupted by phthalates given their known interactions with structurally similar compounds (Schaiff et al. 2006). For steroidogenesis, target genes included *CYP19*, P450 cholesterol side chain cleavage enzyme (*P450scc*), 17β-hydroxysteroid dehydrogenase type 1 (*17*β*-HSD*), and cytochrome P450 1B1 (*CYP1B1*). For trophoblast differentiation, target genes included *PPAR*γ, human chorionic gonadotropin (*HCG*), and the aryl hydrocarbon receptor (*AhR*).

## Materials and Methods

### Study participants

Fifty-four of 148 participants in the Columbia Center for Children’s Environmental Health (CCCEH) with placentas collected at delivery between May 2002 and June 2005 and with both maternal urine samples and medical record data were included this study. Placentas were sampled from 70% (148 of 211) of the 211 CCCEH births over this time; the remainder could not be obtained because of lack of notification of labor onset and other logistical obstacles. CCCEH participants were enrolled through the prenatal clinics at New York Presbyterian and Harlem Hospital Centers in New York City. To be eligible, the woman had to reside in the study area for at least 1 year, receive her first prenatal visit by the 20th week of pregnancy, and be free of diabetes, hypertension, and known HIV and drug or alcohol abuse (Perera et al. 2006; Whyatt et al. 2003). The institutional review boards of Columbia University, the Centers for Disease Control and Prevention (CDC), and the Harvard School of Public Health Human Subjects Committee approved the CCCEH study and substudies. Written informed consent was obtained from all study participants.

### Placenta sampling

The 54 placentas were sampled between 4 min and 2 hr after delivery. Samples of chorionic villi were taken on the fetal side of the placenta. One sample was taken from the inner region proximal to the umbilical cord insertion point, and one from the outer region closer to the edge, yielding two samples per placenta. Methods and rationale for the sampling scheme are described in detail elsewhere (Adibi et al. 2009b). We attempted to control for within-placenta variability by collecting one sample from the inner and one from the outer region of each placenta. Care was taken in dissection to maximize the amount of villous tissue in the sample and to avoid membrane contamination as well as decidua contamination. Samples were preserved in RNAlater (Ambion, Austin, TX) to stabilize the RNA and stored at − 80°C.

### RNA analysis

Total RNA was isolated from approximately 300 mg of tissue using the RNeasy Midi Kit (Qiagen, Valencia, CA). Genomic DNA contamination in the sample was minimized with a DNase digestion step (Rozen and Skaletsky 2000). Total RNA was measured by determining absorbance at 260 nm using an Ultrospec 2100 pro ultraviolet/visible spectrophotometer (GE Healthcare, Piscataway, NJ). RNA purity was assessed by the ratio of absorption at 260 nm to 280 nm and visually by agarose gel electrophoresis. Approximately 3 μg total RNA was used in a reverse transcription (RT) reaction to synthesize cDNA using the SuperScript First-Strand Synthesis System from Invitrogen (Carlsbad, CA). Finally, quantitative real-time polymerase chain reaction (qPCR) was used to quantitate mRNA levels in each sample for individual genes. Ribosomal RNA from *18S* was selected as a housekeeping gene to serve as an internal control for quantity and quality of cDNA going into the RT reaction, based on the results of a previous analysis (Adibi et al. 2009b). All samples were analyzed using the ABI Prism 7500 Sequence Detection System (Applied Biosystems, Foster City, CA). Cycling conditions were the same for seven transcripts (*18S*, *CYP19*, *PPAR*γ, *AhR*, *CYP1B1*, *17*β*-HSD*, *HCG*): 95.0°C for 5 min for activation of the enzyme, 95.0°C for 30 sec for denaturation and 60.0°C for 1 min for annealing/extension for 40 cycles, followed by a dissociation step. Cycling conditions for *P450scc* were the same except the annealing/extension was carried out at 55.0°C for 1 min. Forward and reverse primers (Sigma, St. Louis, MO) were either designed by Primer3 (Rozen and Skaletsky 2000) or selected from the literature and referenced by a PubMed Identifier (PMID; Table 1). Each reaction used 2 μL or 90 ng cDNA assuming 90% efficiency of the cDNA synthesis reaction, forward and reverse primers at optimized concentrations, and SYBR Green PCR Master Mix kit (Applied Biosystems, Foster City, CA) for a total reaction volume of 25 μL.

### Specificity and quantitation

Each sample was run in duplicate, and values not falling within 50% of their mean were rerun. Specificity of the PCR product was evaluated using the melting curve generated at the end of amplification and by running a 2% agarose gel to visualize the PCR product. Absolute quantitation of mRNA concentration in the original sample was achieved using a standard curve generated for each batch. Each standard curve included two nontemplate controls and eight serial dilutions covering the range of 10^3^ to 10^7^ molecules/μL. The standards for each gene were prepared as described previously (Bhat and Epelboym 2004). The *R*^2^ for the standard curve was between 0.98 and 1.00; the plate was rerun if it was < 0.95. The ratio of target gene mRNA molecules to 18S mRNA molecules was calculated for each.

### Phthalate metabolite measurements

Maternal urine samples, collected in the early third trimester (*n* = 54), were analyzed for the four DEHP metabolites mono(2-ethylhexyl) phthalate (MEHP), mono(2-ethyl-5-oxohexyl) phthalate (MEOHP), mono(2-ethyl-5-hydroxyhexyl) phthalate (MEHHP), and mono-2-ethyl-5-carboxypentyl phthalate (MECPP); the DnBP metabolite mono-*n*-butyl phthalate (MnBP); the DiBP metabolite monoisobutyl phthalate (MiBP); and the BBzP metabolite monobenzyl phthalate (MBzP) at the CDC. Because of the high correlations among MEOHP, MEHHP, and MECPP (Spearman correlation *r* = 0.96–0.98), we limited our analysis to MEOHP. To represent total DEHP urinary concentration, we summed the four DEHP metabolites (MEHP, MEOHP, MEHHP, MECPP) in nanomoles per liter (∑DEHP).

The analytical approach involved enzymatic deconjugation of the phthalate metabolites from their glucuronidated form, solid-phase extraction, separation with high-performance liquid chromatography, and detection by isotope-dilution tandem mass spectrometry (Kato et al. 2005). To monitor for accuracy and precision, each analytical run included the unknown samples together with calibration standards, reagent blanks, and quality control materials of high and low concentration. The limits of detection (LODs) were (in nanograms per milliliter) MEHP, 0.9; MEOHP, 0.45; MEHHP, 0.32; MECPP, 0.25; MiBP, 0.26; MnBP, 0.40; and MBzP, 0.11. Concentrations below the LOD were set to one-half the LOD for statistical analysis. Specific gravity was measured at CDC using a PAL 10-S hand-held refractometer (Atago, Bellevue, WA). Urinary concentrations were adjusted for specific gravity using a modification of the formula by Hauser et al. (2004): *P*_c_ = *P* × [(1.016 − 1)/(SG − 1)], where *P*_c_ is the specific-gravity–corrected phthalate concentration, *P* is the observed phthalate concentration, and SG is the specific gravity of the urine samples.

### Statistical analysis

Gene expression values and phthalate metabolite concentrations were log transformed to approximate a normal distribution. *Z*-scores were calculated individually for each gene to put them on the same scale. The *Z*-score, also called the standard score, uses the population mean and standard deviation to standardize or normalize sample values so that they share a common underlying distribution. *Z*-scores were modeled in two groups by functional pathways, steroidogenesis and trophoblast differentiation. Spearman correlation coefficients were used to estimate pairwise associations among gene transcripts. Variance component analysis was used to evaluate between- versus within-placenta variability.

Multivariate mixed effects models were used to estimate associations between gene expression and specific gravity–adjusted phthalate metabolite concentrations (Fitzmaurice et al. 2004; Verbeke and Molenberghs 2000). Gene expression *Z*-scores for multiple genes within a pathway were modeled as a correlated vector of eight responses within each placenta for the steroidogenesis pathway (i.e., two samples per placenta and four target genes measured on each sample) and six responses for trophoblast differentiation (i.e., two samples per placenta and three target genes measured on each sample) as a function of phthalate concentrations and other covariates. The mixed effect model approach allows for the analysis of the expression of each gene separately while taking into account differences between genes within a pathway, and simultaneously adjusting for the correlation between the two samples and three to four target genes measured on a single placenta. We assumed equal correlation between any two responses measured within a single placenta. We grouped the gene transcripts by pathway to most efficiently use information collected from each placenta and to increase our statistical power to detect associations with phthalate exposures. Urinary phthalate concentrations were considered both as log-transformed continuous measures (assuming linearity of dose response) and as grouped into quintiles to avoid the assumption of linearity. We used the fitted mixed effect model to calculate the predicted mean *Z*-scores and standard errors for each quintile of phthalate exposure, in order to illustrate dose–response relationships. The assumption that the dose–response pattern was the same across all target genes within a pathway was assessed by including additional interaction terms between type of gene and quintile of exposure; tests of interactions significant at *p* < 0.05 suggest a lack of agreement in dose–response patterns.

We evaluated model fit and potential confounding by sampling characteristics and other variables (season, demographic characteristics, maternal size and adiposity, smoking status, maternal health, pregnancy history, and fetal sex). Covariates other than specific-gravity–adjusted phthalate exposure levels and gene (categorical variable with a level for each gene in pathway) were retained in the model if significant at *p* < 0.05. In our final models, we controlled for qPCR plate, season of delivery, and level of education. In the steroidogenesis models, we also controlled for year of delivery. In the trophoblast differentiation model, we also controlled for mother’s ethnicity, net weight gain, and history of hypertension. Statistical significance was defined by a (two-sided) *p*-value ≤ 0.05. SAS version 9.1 (SAS Institute Inc., Cary, NC) software was used to conduct all analyses.

## Results

The 54 women included in our study were a subset of CCCEH participants similar demographically to those in the parent study except with a larger proportion of Dominican women (83% vs. 74%) and a higher percentage of participants with a high school degree or GED (65% vs. 37%) (Table 2). The distributions of the urinary phthalate metabolites were similar to those reported in a larger sample from the same cohort (Table 3) (Adibi et al. 2008).

The median yield of total RNA per placenta biopsy was 62 μg (mean ± SE = 72 ± 5 μg). Analytic gels showed two distinct bands at 18S and 28S with minimal signs of degradation. Of the 108 biopsies from 54 placentas, two samples had insufficient RNA/cDNA at the time of qPCR analysis for HCG, and one for 17β-HSD. One RNA/cDNA sample was missing at the time of analysis of *18S* (housekeeping gene), which is the reason for the single missing value across all remaining transcripts (Table 4). The ranking of transcripts by median message level (mRNA/3 μg total RNA) was as follows: *18S rRNA* (9.80 × 10^5^), *HCG* (4.30 × 10^5^), *P450scc* (3.94 × 10^4^), *CYP19* (3.30 × 10^4^), *17*β*-HSD* (7.47 × 10^3^), *PPAR*γ (3.56 × 10^3^), *AhR* (5.03 × 10^2^), and *CYP1B1* (1.02 × 10^2^).

Between-placenta variability was higher than within-placenta variability for *CYP19* (62% vs. 38%) and *AhR* (59% vs. 42%). For four other transcripts, the between-placenta variability was slightly lower than 50% (*PPAR*γ, 47%; P450scc, 40%; *17*β*-HSD*, 41%; *HCG*, 46%), and was 30% for *CYP1B1*. This was consistent with our previous analysis that demonstrated high within-placenta variability, which was mitigated by controlling for location within the chorionic plate (Adibi et al. 2009b). Transcripts were significantly correlated within the pathway groupings (Table 4).

**Figure 1:**
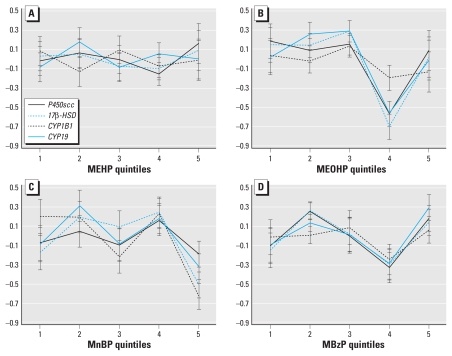
Quintile plots of estimated mean *Z*-scores (± SE) of gene transcripts in the placental steroidogenesis pathway in relation to maternal urinary concentrations of MEHP (*A*), MEOHP (*B*), MnBP (*C*), and MBzP (*D*). *C* depicts a significant association between MnBP quintiles and steroidogenic gene expression (*p* = 0.001) and significantly different slopes among genes in relation to MnBP (*p* = 0.03).

We fitted mixed effects models both with and without the assumption of linearity of phthalate exposure effects to evaluate associations with gene expression levels within each pathway (Tables 5 and 6). In the case of steroidogenesis, we found no significant associations of the phthalate metabolite levels with target genes in the models using log concentration levels (i.e., assuming linearity) (Table 5). We found a significant difference across quintiles of MnBP exposure (*p* = 0.001) (Table 6), with a trend toward a U-shaped dose–response pattern (Figure 1C). The Pearson correlation between the *Z*-score and the log-transformed gene expression value (ratio of target gene to 18S RNA) was 1.00. Even though *Z*-scores are not typically used in the presentation of qPCR data, they should be interpreted as a direct proxy for the mRNA levels measured in the sample.

**Figure 2:**
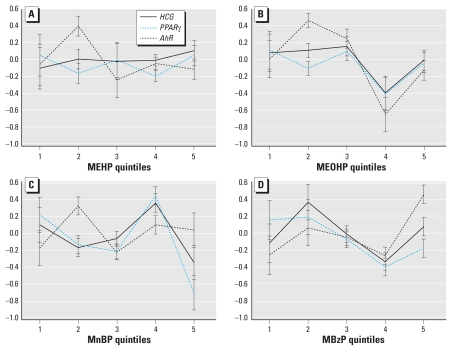
Quintile plots of estimated mean *Z*-scores (± SE) of gene transcripts in the trophoblast differentiation pathway in relation to maternal urinary concentrations of MEHP (*A*), MEOHP (*B*), MnBP (*C*), and MBzP (*D*). *B–D* depict significant associations between MEOHP (*p* = 0.002), MnBP (*p* = 0.004), and MBzP (*p* = 0.01) quintiles and gene expression; *C* and *D* depict significantly different slopes among genes in relation to MnBP (*p* = 0.03) and MBzP (*p* = 0.01).

For the trophoblast differentiation pathway, higher levels of urinary metabolite concentrations were associated with significantly lower levels of gene expression for all five phthalate metabolites and ∑DEHP (Table 5). We also observed differences in gene expression across the quintiles of exposure for all metabolites except MEHP (Table 6, Figure 2). Although the trend was generally inverse, there was a suggestion of a U-shaped curve for all metabolites (Figure 2).

For most phthalate metabolites, additional tests of interaction between individual gene and metabolite levels were not significant and thus supported the assumption of a similar pattern of dose response for genes within the same pathway. That is, the shift in *Z*-scores for each quintile of exposure level did not depend on which of the genes we measured. However, there were isolated exceptions. For the steroidogenesis pathway, we found a significant difference in dose–response patterns of target genes across levels of MnBP exposure (*p* = 0.03). For the trophoblast pathway, we found a difference in dose–response patterns across target genes for MnBP (*p* = 0.03) and MBzP (*p* = 0.01; see Figure 2C,D).

## Discussion

In a sample of 54 Dominican and African-American women in New York City, urinary phthalate metabolite concentrations were associated with placental biomarkers of gene expression in two pathways, steroidogenesis and trophoblast differentiation.

The consistent decreases in placental gene expression at the higher quintile concentrations of phthalate metabolites may mean that effects are concentrated at the higher doses or that women in the upper exposure quintiles are more susceptible to placental insults for other reasons that are correlated with phthalate exposures that we were unable to control for. This cohort, which is characterized by low income and high social disadvantage, has significantly higher urinary concentrations of DnBP and DiBP metabolites compared with other pregnant women in the U.S. general population and in another U.S. multicenter pregnancy cohort (Adibi et al. 2009a). Given that high exposures to chemicals may be accompanied by poor nutrition and co-exposures to other chemicals and other physical and psychosocial stress, these women and their fetuses may be in an especially high category of risk (Rauh et al. 2004).

Results differed slightly when we applied modeling strategies that assume linearity in the dose response versus those that do not. Inspection of the associations by level of exposure shows little evidence of linearity with most of the metabolites. Dose–response relationships within these pathways may be nonmonotonic, which would be expected given the nature of transcriptional regulation of nuclear receptors and endocrine signals and specifically with regard to the behavior of phthalates and other endocrine-disrupting compounds (Andrade et al. 2006; Li et al. 2007; Welshons et al. 2003).

There is no consensus on the molecular mechanism of phthalate actions, especially with regard to the steroidogenic effects (Feige et al. 2007; Hallmark et al. 2007; Wilson et al. 2004). Some data show effects at the level of mRNA transcription that are consistent with protein levels (Lovekamp and Davis 2001; McKinnell et al. 2005; Wilson et al. 2004), whereas others show effects at the level of protein expression but not transcription (Boberg et al. 2008; Lambrot et al. 2009). Attempts to recapitulate the steroidogenic effects of MEHP and MnBP in rodents using human testis explants have produced conflicting results (Hallmark et al. 2007; Lambrot et al. 2009), which could be due to differences in experimental systems and/or species. The association that we measured between phthalates and placental steroidogenesis was present only in the quintile models with MnBP. Interestingly, there is some evidence in humans that fetal testicular steroidogenesis and placental steroidogenesis may be linked (Akre et al. 2008).

The trophoblast differentiation pathway (*PPAR*γ, *HCG*, *AhR*) was of interest to us as a well-studied PPARγ-mediated pathway in the human placenta (Tarrade et al. 2001). In fact, HCG is a marker of syncytium formation and is used as an indicator of placental function in clinical tests (Lepage et al. 2003; Yang et al. 2003). Beyond its role in xenobiotic metabolism, AhR is also believed to be a regulator of estrogen metabolism, vascularization, and hypoxic responses in the placenta (Detmar et al. 2008; Huuskonen et al. 2008). Trophoblast differentiation is most important early in pregnancy when the placenta is initially being constructed and assuming its spatial and physiologic orientation with respect to the fetus. Throughout pregnancy, a population of progenitor trophoblasts persists and undergoes renewal and differentiation (Tarrade et al. 2001). Toward late pregnancy, there is a shift toward higher proportions of syncytiotrophoblasts where PPARγ is localized (Borel et al. 2008; Rodie et al. 2005). Our ability to detect an association between phthalate metabolite urinary concentrations and trophoblast differentiation could mean that there was a disruption of this late-stage process. There may also have been effects on trophoblast differentiation and placental development early in pregnancy that were mirrored in these biomarkers measured at term.

It is difficult to draw conclusions with a limited set of gene targets per pathway. These targets and their relationship to phthalate metabolites should be pursued further by including additional gene targets and posttranscriptional markers. Nonetheless, correlations of maternal and fetal exposures with transcription may provide valuable information even if it is not possible to extrapolate to posttranscriptional phenotype. In most cases, our assumption that genes within a pathway were expressed in a parallel dose response was supported by the data.

In summary, we applied biomarkers of mRNA transcription in human placental tissue to test hypotheses on the association of prenatal phthalate exposure with placental development and function. Metabolites of DEHP, DnBP, DiBP, and BBzP were significantly associated with the joint expression of three gene targets known to be involved in trophoblast differentiation (*PPAR*γ, *AhR*, *HCG*). These associations were robust to linear and nonlinear modeling strategies and to adjustment for urinary dilution. We did not detect robust associations of phthalate metabolites with the joint expression of four gene targets in the steroidogenic pathway (*CYP19*, *17*β*-HSD*, *P450scc*, *CYP1B1*). This may suggest a null association of phthalates with placental steroidogenesis, or it may suggest lack of sensitivity in our methodology. It may also indicate that the model of phthalate-induced perturbation of steroidogenesis well described in rodent models cannot be directly translated to humans. In either case, we offer a novel approach to study the effects of endocrine-disrupting compounds on placental function.

## Figures and Tables

**Table 1:** Primer sets for qPCR analysis.

Gene	Direction	Primer sequence	Base pairs	Reference
*CYP19*	Forward	ATACCAGGTCCTGGCTACTG	249	PMID 11745463^a^
	Reverse	TCTCATGCATACCGATGCACTG		
*PPAR*γ	Forward	GCTGTGCAGGAGATCACAGA	225	Designed in Primer3
	Reverse	GGGCTCCATAAAGTCACCAA		
*AhR*	Forward	ACATCACCTACGCCAGTCGC	101	PMID 12520072^b^
	Reverse	TCTATGCCGCTTGGAAGGAT		
*CYP1B1*	Forward	ACCGTTTTCCGCGAATTC	196	PMID 12040753^c^
	Reverse	GTACGTTCTCCAAATCCAGCC		
*P450scc*	Forward	CCTGCAGTGGCACTTGTATG	418	PMID 11997174^d^
	Reverse	GGTCATCTCTAGCTCAGCGA		
*17*β*-HSD type 1*	Forward	GCCGCGTGGACGTGCTGGTGTGTAAC	201	PMID 12242730^e^
	Reverse	CCATCAATCCTCCCACGCTCCCGG		
*HCG*	Forward	TCCCACTCCACTAAGGTCCAA	106	PMID 15081642^f^
	Reverse	CCCCATTACTGTGACCCTGTT		
*18S rRNA*	Forward	CGGCTACCACATCCAAGGAA	187	PMID 14583453^g^
	Reverse	GCTGGAATTACCGCGGCT		

a Miyoshi et al. 2001.

b Lin et al. 2003.

c Nishimura et al. 2002.

d Yu et al. 2002.

e Koh et al. 2002.

f Pidoux et al. 2004.

g Vissac-Sabatier et al. 2003.

**Table 2:** CCCEH sample characteristics (*n* = 54).

Characteristic	Value
Gestational age (weeks)^a^	39.5 ± 1.6
Birth weight (g)^b^	3,324 ± 466
Maternal age (years)	26.1 ± 4.5
Gestational age^a^	
< 37 weeks	3 (6)
37–41 weeks	45 (88)
> 41 weeks	3 (6)
Race/ethnicity	
Dominican	45 (83)
African American	9 (17)
Marital status	
Never married	35 (65)
Widowed, divorced, separated	4 (7)
Married	15 (28)
Education	
Less than high school	6 (11)
High school or GED	35 (65)
More than high school	13 (24)
Parity	
0 live births	21 (39)
1 live birth	19 (35)
> 1 live births	14 (26)
Cesarean section^c^	14 (27)
Body mass index^d^	
≤ 24.9	29 (54)
> 24.9 and ≤ 29.9	12 (22)
> 29.9	10 (19)
Female sex, newborn (%)	26 (48)
Season	
Summer	13 (24)
Fall	8 (15)
Winter	17 (31)
Spring	16 (30)
Year of delivery	
2002	8 (15)
2003	13 (24)
2004	14 (26)
2005	19 (35)

Values are mean ± SD or no. (%).

a Three missing for gestational age.

b Three missing for birth weight.

c Two missing for delivery method.

d Three missing for body mass index.

**Table 3:** Distribution of maternal urinary phthalate metabolites (ng/mL; *n*= 54).

Measure	MEHP	MEOHP	∑DEHP metabolites^a^	MnBP	MiBP	MBzP
Geometric mean	5.5	16.5	279.8	34.6	10.4	20.4
Quintiles, specific-gravity adjusted						
Quintile 1	≤ 2.2	≤ 10.2	≤ 112.2	≤ 19.6	≤ 6.2	≤ 5.7
Quintile 2	2.3–4.8	10.3–15.0	112.3–221.1	19.7–37.9	6.3–11.1	5.8–15.7
Quintile 3	4.9–9.6	15.1–26.0	221.2–371.5	38.0–52.8	11.2–14.1	15.8–32.1
Quintile 4	9.7–18.8	26.1–47.5	371.6–735.2	52.9–73.0	14.2–24.4	32.2–88.9
Quintile 5	> 18.8	> 47.5	> 735.2	> 73.0	> 24.4	> 88.9

a Sum of MEHP, MEOHP, MEHHP, and MECPP, in nanomoles/liter.

**Table 4:** Mean values of log-transformed gene transcripts and their corresponding mean *Z*-scores and Spearman correlations (95% confidence interval) between placental mRNA levels, adjusted for 18S mRNA, grouped by common pathway (*n* = 54 placentas).

	Gene transcript			
Measure	*CYP19*^a^	*17*β*-HSD*^b^	*P450scc*^a^	*CYP1B1*^b^
Steroidogenesis pathway				
Mean ± SD, log transformed	−3.4 ± 3.3	−4.2 ± 1.6	−2.4 ± 2.0	−8.4 ± 1.6
Mean *Z*-score ± SD	0.01 ± 1.1	−0.02 ± 1.2	−0.01 ± 1.2	−0.02 ± 1.2
Spearman correlation (95% CI)				
*CYP19*	1.00	0.75 (0.65–0.82)	0.85 (0.79–0.90)	0.62 (0.49–0.72)
*17*β*-HSD*		1.00	0.87 (0.81–0.91)	0.69 (0.57–0.78)
*P450scc*			1.00	0.63 (0.50–0.73)
	*PPAR*γ^a^	*AhR*^a^	*HCG*^b^	

Trophoblast differentiation pathway				
Mean ± SD, log transformed	−5.3 ± 2.1	−7.1 ± 2.5	−0.9 ± 2.7	
Mean *Z*-score ± SD	−0.04 ± 1.1	0.02 ± 1.1	0.01 ± 1.1	
Spearman correlation (95% CI)				
*PPAR*γ	1.00	0.41 (0.24–0.56)	0.83 (0.75–0.88)	
*AhR*		1.00	0.68 (0.56–0.77)	

a One missing (*n* = 107).

b Two missing (*n* = 106).

**Table 5:** Associations between pathway-specific placental gene expression and maternal urinary phthalate metabolites adjusted for specific gravity (log-transformed), using a linear model approach.

	β-Coefficient (SE), *p*-value	
Phthalate metabolite	Steroidogenesis: *CYP19*, *17*β*-HSD*, *P450scc*, *CYP1B1*^a^	Trophoblast differentiation: *PPAR*γ, *AhR*, *HCG*^b^
MEHP	0.01 (0.12), *p* = 0.90	−0.15 (0.06), *p* = 0.02
MEOHP	−0.04 (0.13), *p* = 0.74	−0.28 (0.06), *p* < 0.0001
∑DEHP metabolites^c^	−0.01 (0.13), *p* = 0.96	−0.19 (0.08), *p* = 0.03
MnBP	−0.19 (0.16), *p* = 0.25	−0.16 (0.08), *p* = 0.05
MiBP	−0.11 (0.14), *p* = 0.46	−0.21 (0.09), *p* = 0.02
MBzP	0.03 (0.09), *p* = 0.78	−0.14 (0.06), *p* = 0.02

a *n* = 54, adjusted for gene, qPCR batch (*CYP1B1*), year of delivery, season of delivery, and level of education.

b *n* = 54, adjusted for gene, qPCR batch (*AhR*, *PPAR*γ), season of delivery, level of education, net weight gain, mother’s ethnicity, and history of hypertension.

c The square root of specific gravity was included as an independent term in the model.

**Table 6:** Associations between pathway-specific placental gene expression and maternal urinary phthalate metabolites, using a quintile model approach.

	β-Coefficient (SE), *p*-value									
	Steroidogenesis: *CYP19*, *17*β*-HSD*, *P450scc*, *CYP1B1*^a^					Trophoblast differentiation: *PPAR* γ, *AhR*, *HCG*^b^				
Phthalate metabolite	Quintile 2	Quintile 3	Quintile 4	Quintile 5	*p*-Value^c^	Quintile 2	Quintile 3	Quintile 4	Quintile 5	*p*-Value^c^
MEHP	0.26 (0.35)	−0.28 (0.47)	−0.13 (0.43)	0.30 (0.42)	0.29	0.02 (0.29)	−0.15 (0.26)	−0.58 (0.30)	−0.43 (0.26)	0.22
MEOHP	−0.50 (0.38)	−0.34 (0.28)	−0.95 (0.43)*	−0.03 (0.39)	0.06	−0.59 (0.31)	−0.18 (0.24)	−0.92 (0.25)**	−0.83 (0.23)**	0.002
∑DEHP metabolites^d^	−0.36 (0.36)	0.03 (0.42)	−0.40 (0.41)	−0.01 (0.47)	0.54	−0.13 (0.30)	−0.90 (0.33)*	−0.90 (0.28)**	−0.92 (0.27)**	0.02
MnBP	0.72 (0.39)	0.48 (0.46)	0.83 (0.38)*	−0.61 (0.41)	0.001	0.07 (0.29)	−0.52 (0.26)*	0.33 (0.28)	0.57 (0.21)**	0.004
MiBP	0.14 (0.53)	0.35 (0.61)	−0.18 (0.45)	−0.22 (0.44)	0.71	0.43 (0.24)	0.19 (0.36)	−0.92 (0.20)**	−0.44 (0.23)	0.0002
MBzP	0.14 (0.49)	0.09 (0.42)	−0.14 (0.43)	0.12 (0.35)	0.89	0.04 (0.30)	−0.52 (0.27)	−0.67 (0.24)**	−0.42 (0.26)	0.01

a *n* = 54, adjusted for gene, qPCR batch (*CYP1B1*), year of delivery, season of delivery, and level of education.

b *n* = 54, adjusted for gene, qPCR batch (*AhR*, *PPAR*γ), season of delivery, level of education, net weight gain, mother’s ethnicity, and history of hypertension.

c The type 3 test of fixed effects, a four degrees of freedom test of whether there is any difference among the 5 groups. Quintile 1 is the referent group.

d The square root of specific gravity was included as an independent term in the model.

* *p* = 0.05,

** *p* = 0.01; quintile 1 is the referent group in the regression models.
