# Correction: Transcriptional Biomarkers of Steroidogenesis and Trophoblast Differentiation in the Placenta in Relation to Prenatal Phthalate Exposure

**DOI:** 10.1289/ehp.0900788-erratum

**Published:** 2010-03-01

Adibi et al. reported an error in Table 6 of “Transcriptional Biomarkers of Steroidogenesis and Trophoblast Differentiation in the Placenta in Relation to Prenatal Phthalate Exposure” [Environ Health Perspect 118:291–296 (2010)]. In Quintile 5 of Trophoblast Differentiation, the value for MnBP should be –0.57 rather than 0.57.

